# Lung Segmentectomy in NSCLC Surgery

**DOI:** 10.3390/life13061284

**Published:** 2023-05-30

**Authors:** Alberto Salvicchi, Simone Tombelli, Giovanni Mugnaini, Alessandro Gonfiotti

**Affiliations:** 1 Thoracic Surgery Unit, Careggi University Hospital, 50134 Florence, Italy; 2 Department of Experimental and Clinical Medicine, University of Florence, 50134 Florence, Italy

**Keywords:** segmentectomy, lung surgery, NSCLC, lobectomy, early-stage, lung cancer, sublobar

## Abstract

Current guidelines recommend surgery for early-stage non-small cell lung cancer (NSCLC). The standard treatment for patients with cT1N0 NSCLC has been lobectomy with lymph-node dissection, with sublobar resection used only in patients with inadequate cardio-respiratory reserve, with poor performance status, or who are elderly. In 1995, the Lung Cancer Study Group published the results of a randomized, prospective trial demonstrating the superiority of lobectomy compared with sublobar resection. From then on, wedge resection and segmentectomy were reserved exclusively for patients with poor functional reserve who could not tolerate lobectomy. Therefore, the exact role of segmentectomy has been controversial over the past 20 years. Recently, the randomized controlled trial JCOG0802/WJOG4607L demonstrated that segmentectomy was superior to lobectomy in patients with stage IA NSCLC (<2 cm and CTR < 0.5) in terms of both overall-survival and post-operative lung function. Based on these results, segmentectomy should be considered the standard surgical procedure for this patient group. In 2023, the randomized phase III CALGB 140503 (Alliance) trial demonstrated the efficacy and non-inferiority of sublobar resection, including wedge resection, for clinical stage IA NSCLC with tumor diameter of < 2 cm. This article is a narrative review of the current role of segmentectomy in lung cancer treatment and summarizes the most relevant studies in this context.

## 1. Introduction

The well-known prospective randomized multi-institutional trial conducted by Ginsberg and Rubenstein and the Lung Cancer Study Group (LCSG) in 1995 demonstrated that lobectomy and systematic lymph node dissection is the standard treatment for early-stage non-small-cell lung cancer (NSCLC) [1] cT1N0. The results of this study showed that sublobar resections had a local recurrence rate three times higher than that of lobectomy and a 50% higher cancer-related mortality rate.

However, in the last two decades several studies have suggested more and more often that sublobar lung resections (wedge resections, but especially anatomic segmentectomies) could be carried out not only in high-comorbidity patients who cannot tolerate lobectomy [2].

From a technical point of view, sublobar resections include both wedge resection and segmentectomy, and the surgical difficulties differ considerably between the two types: wedge resection is the non-anatomical removal of lung tissue containing the tumor, while segmentectomy involves suturing and section of the arteries, veins, and bronchi associated with the lung segment.

In recent years, the detection of small nodules or ground-glass opacities suggestive of tumor has increased due to CT low-dose screening programs and improved diagnostic modalities, so that indication for sublobar resections became more common and some surgeons began to practice them, even though there were no randomized trials. The turning point came in 2022 and early 2023 with the publication of the results of the two most important randomized controlled trials of recent years in this field, the JCOG0802/WJOG4607L study and the CALBG/Alliance 140503 study (Table 1). Both demonstrated the non-inferiority of sublobar resection on oncological outcomes in terms of overall survival (OS), disease-free survival (DFS), and recurrence rate (RR) [3,4]. Results of the phase III Alliance trial, recently published, showed a 5-year disease-free survival of 63.6% for patients with NSCLC tumors up to 2 cm who underwent a sublobar resection, compared with 64.3% for those who underwent a lobectomy (HR = 1.01). These data allow us to state that sublobar resections, and more specifically anatomical segmentectomies, could become the standard of care for patients with peripheral cT1aN0 NSCLC (≤2 cm) without metastases to major hilar and mediastinal lymph nodes. Anatomic sublobar resections could also play an important role in terms of post-operative pulmonary functions [5]. 

We think that, in the near future, segmentectomy will play an increasingly important role, due also to the several ongoing trials that are examining screening for NSCLC and hence the increased possibility of diagnosis of early-stage NSCLC. 

The aim of this narrative review is to outline the results and outcomes of the most-recent trials and to highlight surgical indications for this technique. 

## 2. Materials and Methods

This narrative review was performed by aggregating the current literature on surgical lung segmentectomy with a focus on its role in early-stage NSCLC, overall survival, and disease-free survival. We analyzed the references available in English published on the PubMed platform using the following terms in the search field: Lung segmentectomy, NSCLC surgery, NSCLC treatment.

## 3. Surgical Strategies

Since the first segmental resection for lung cancer described by Jenkis et al. [6], the debate has focused on finding the correct indications for sublobar resections to achieve significant outcomes in terms of oncological radicality and OS. Sublobar resections, including either wedge and anatomical resection, may be indicated in two different scenarios:

- Patients with several comorbidities and limited functional reserve that contraindicate lobectomy, so as to allow them to be eligible for surgical treatment in early-stage NSCLC while accepting the compromise of a lower long-term survival. This class of patients is also influenced by the surgical technique chosen, with better post-operative recovery with minimally invasive techniques such as video-assisted (VATS) or robot-assisted (RATS) thoracotomy.
- In the case of early diagnosis, following the criteria indicated below, even for patients without comorbidities.

As also indicated by the 2023 NCCN guidelines for NSCLC [7], sublobar resections should be performed in the following case: a single tumor located in the outer third of the parenchyma, NSCLC suspected, clinical stage IA [8], size less than 2 cm in its largest diameter, C/T ratio < 0.5 evaluated using the lung window at CT scan, no lymphnode metastasis. 

### 3.1. Nodule Localization

A crucial point in assessing the feasibility of sublobar resections, both wedge and anatomical, is the size and location of the lesion, which must be a single peripheral nodule, smaller than 2 cm in its largest diameter (considering both the solid component and the associated GGO component). A peripheral lesion is a nodule whose center is in the outer third of the lung parenchyma. This is so that the resection can be technically executable with adequate oncological margins. This type of resection is therefore not applicable for the centroparenchymal nodules.

### 3.2. Consolidation/Tumor Ratio

With the increasing use of imaging techniques in diagnostic examinations and the availability of low-dose CT devices, the diagnosis of early-stage lung lesions has increased. Based on radiological findings, evaluated using the lung window at CT scan, lung lesions can be classified as follows: pure ground glass nodules (GGN), part-solid nodules, and pure solid nodules. GGO is a descriptive term for a nonspecific radiologic finding that refers to an area of increased attenuation in the lung without blockage of the underlying pulmonary vessels or bronchial structures, whereas in pure solid nodules these underlying structures are no longer visible. In non-mucinous adenocarcinomas of the lung, these lesions (GGO vs. solid opacities) are associated with a lepidic or invasive pattern. This correlation is not absolute and several studies have been performed to discover predictive elements based on radiological finding [9,10,11,12,13]. The pathological patterns of growth of sub-solid nodules, including pure and partially solid GGN types and pure solid lesions, are obviously different, leading to different prognoses. As expected, sub-solid nodules with the GGO component have a good prognosis; conversely, pure solid lesions are thought to have high invasiveness with a worse prognosis [10,11]. The results of the JCOG0201 prospective study [13], which showed a consolidation/tumor ratio (CTR) < 0.5 (5-year relapse-free survival 95.9%) [14] as an evaluation criterion for non-invasive lung adenocarcinoma <2.0 cm with a specificity of 98.7% (95% CI: 93.2–100.0%), appear important in this context and this criterion could be used to radiologically define early adenocarcinoma of the lung. CTR was defined as the ratio of the maximum consolidation diameter (C) divided by the maximum tumor diameter (T), which was determined digitally based on the CT scan findings (using the lung window).

### 3.3. Oncological Margins

The main criticism of sublobar resection (especially in complex segmentectomies) is the smaller extension and amplitude of the parenchymal margins than in a standard lobectomy [15]. For this reason, nodule size and location are the main criteria to be analyzed. Sublobar resections such as anatomical segmentectomies and wedge resection should achieve parenchymal resection margins ≥ 2 cm or bigger than the size of the nodule [7]. The correct distance between the tumor margin and the resection margin should be closely examined in the pre-operative period by evaluating all CT scans (i.e., axial, sagittal, coronal views) including, if possible, 3D reconstruction [16] and intra-operatively confirmed with a frozen section. When required, surgical margins have to be widened to grant the best oncological outcome. 

### 3.4. Complex and Simple Segmentectomy

A proper evaluation of the segmental anatomy of the lung is essential for planning the procedure. Although each segment has a different shape, they can generally be considered pyramidal, with the apex pointing toward the hilum. In the CT study, the segments can be identified by following the bronchial and vascular branches, flowing up and down the axial sections. Compared with the bronchial tree branches, evaluation of arterial and venous branches may be more complex because of their anatomic variability and the fact that more arteries may spray the same segment, whereas more veins may provide drainage. From a surgical perspective, anatomic segmentectomies are currently divided into simple and complex procedures [17]. Simple segmentectomies include resection of the apical segment of the lower lobe (both right and left), lingual segmentectomy, or left superior segmentectomy. Complex segmentectomies are anatomic sublobar resections that include segments other than those previously mentioned (Table 2). From a technical perspective, even when defined as “simple,” these anatomical structures are not always easy to identify and isolate. Compared to standard lobectomies, they require a longer learning curve. We could define a complex segmentectomy as the one that requires resection of more than one intersegmental plan (i.e., S2 or the S1–S2 bisegments in the right upper lobe) and, as referenced by many authors, are considered as a challenging resection even for a certified thoracic surgeon, also because the broncovascular structures are located deeper [18]. The complexity of this procedure is demonstrated by higher Prolonged Air Leak (PAL) rates and narrower surgical resection margins. Considering the previous paragraph, the correct location of the nodule is crucial in order to ensure adequate resection margins, especially for complex segmentectomies [19]. [Table 1].

### 3.5. Lymphadenectomy

As described previously, sublobar resections can be indicated for selected patients with clinical stage IA NSCLC, so pre-operative examination with CT and PET-CT should exclude nodal involvement. In the event that CT examination shows enlargement of mediastinal lymph nodes or PET-CT examination shows hyperactivity of lymph nodes, endobronchial ultrasound transbronchial needle aspiration (EBUS-TBNA) or mediastinoscopic biopsy must be performed. Segmental lymph node dissection for intra-operative frozen section examination should be performed according to ESTS guidelines, and, as it was recently shown by the JCOG0802 and CALGB trials, absence of metastases in hilar and mediastinal lymph nodes has to be demonstrated as far as possible [20]. The importance of lymphadenectomy and its extension (considering N1 hilar lymph node stations, or N2 mediastinal nodes) are a subject of discussion since lesions that can be applied to this type of resection must be early-stage, node negative. For lobectomies, adequate lymph node dissection, including both hilar and mediastinal nodes, has been shown to be essential to ensure local disease control and to ensure proper staging [21]. In recent years some studies have tried to define this point for sublobar resections [22]. Handa et al. [23], in their multicenter, propensity-score-matched analysis, compared segmentectomies associated with hilar lymphadenectomy with those associated with mediastinal lymphadenectomy and concluded that a well-performed segmentectomy also requires a mediastinal lymphadenectomy, as this procedure allows the harvest of more lymph nodes and provides a more appropriate pathological staging compared to segmentectomies followed only by hilar lymphadenectomy.

### 3.6. Vats and Rats Resections

Sublobar resections and minimally invasive surgery are evolving together as they share a common goal, that is, to perform a proper and oncologically correct resection with an influence on patients’ post-operative quality of life that is as small as possible. Uniportal VATS resections are now performed all around the world with a standardized technique, although the learning curve is a little longer than that of standard VATS resections. In addition, robot-assisted resections are performed worldwide. RATS segmentectomies have similar post-operative outcomes compared to VATS resections in terms of post-operative complications, quality of life, and pain management, without any difference in oncological outcomes or number of lymph node stations resected. Furthermore, in RATS, the better visualization of the hilar structures of the lung segment often allows a more anatomically correct resection to be performed [24]. 

## 4. Outcomes

The first randomized controlled trial comparing oncologic outcomes between lobectomy and segmentectomy was the Lung Cancer Study Group (LCSG) [1], and its results were published in 1995. According to the authors of the study, sublobar lung resection was associated with a statistically significant increase in death and local recurrence. However, this study had some major limitations that were later criticized by other authors, such as the inclusion of tumors up to 3 cm in diameter, the large number of wedge resections in the sublobar group (32.8%), and the nonroutine use of CT in pre-operative evaluation and post-operative follow-up [25].

Several studies have been published by the LCSG, which evaluated the surgical effectiveness and the oncologic feasibility of segmentectomy compared with lobectomy. Okada et al. 2001 [26] tested intentional sublobar resection in comparison to lobectomy in uncompromised patients with cT < 2 cm N0M0 NSCLC. The authors demonstrated that segmentectomy was noninferior to lobectomy in terms of prognosis in patients with early-stage NSCLC, with similar survival in both groups: 5-year overall survival was 85.9% for lobectomy and 83.4% for the sublobar resection (*p* = 0.2778).

Similar results were reported by Landreneau et al. [27] in a large propensity-matching comparison. This study compared segmentectomy with lobectomy for clinical T < 3 cm N0M0 and reported 5-year survival rates of 54% and 60%, respectively (*p* = 0.258), and no differences were observed in terms of rate of locoregional or distant recurrence. Wen et al. [28] hypothesized that surgical segmentectomy might have overall survival and disease-free survival comparable to lobectomy in patients with T1 N0M0 NSCLC. The authors conducted a retrospective study of 369 patients who underwent anatomic segmentectomy (*n* = 90) and lobectomy (*n* = 279) for early-stage NSCLC. The results showed that segmentectomy was noninferior to lobectomy because there were no statistical differences in disease recurrence and 5-year survival between segmentectomy and lobectomy. In addition, segmentectomy was not associated with an increased incidence of serious complications, hospitalization, or mortality. Other publications reported equivalence of oncologic outcomes between segmentectomy and lobectomy. In their systematic review, Ijsseldijk et al. [29] found that overall survival and recurrence-free survival in pT1a NSCLC after segmentectomy were comparable to those of lobectomy. 

Bongiolatti et al. [18], in their retrospective study, demonstrated the feasibility and oncologic appropriateness of simple and complex segmentectomies in the treatment of early-stage lung cancer; the authors reported good results in terms of overall survival (96%) and disease-free survival (78%) and even a low rate of local relapse.

However, there are also studies in the literature that do not confirm the oncologic equivalence between segmentectomy and lobectomy. For example, Zhang et al. [30] claimed that segmentectomy was inferior to lobectomy in terms of survival in stage I NSCLC and that tumor size and age should not be used as criteria for segmentectomy. Zang and colleagues in their meta-analysis study reported that lobectomy was significantly superior to segmentectomy in terms of survival in stage I NSCLC.

Until recently, lobectomy was considered the gold standard for the treatment of early-stage lung cancer, as the only randomized controlled trial was that of Ginsberg in 1995, which confirmed the superiority of lobectomy in terms of overall survival. 

In the last year, two large randomized controlled trials have compared lobectomy with sublobar resection, more specifically, JCOG0802/WJOG4607L [3] and CALGB/ALLIANCE 140503 [4]. The results of these studies have demonstrated the pivotal role of sublobar resection in selected patients with clinical stage IA NSCLC.

The JCOG0802/WJOG4607L trial enrolled 1106 patients with tumor diameter ≤ 2 cm and a consolidation-to-tumor ratio > 0–5 who were randomly assigned to lobectomy (*n* = 554) or segmentectomy (*n* = 552). The primary end point was overall survival, but post-operative respiratory function (6 months and 12 months), recurrence-free survival, hospital stay, duration of surgery, and adverse events were also assessed. In contrast to Ginsberg’s results, JCOG0802 was the first and only randomized trial to demonstrate the superiority of segmentectomy over lobectomy in terms of overall survival for early-stage NSCLC. Median follow-up was 7.3 years, 5-year overall survival was 94.3% for segmentectomy versus 91.1% for lobectomy, and 5-year recurrence-free survival was 88% and 87.9%, respectively. It should be added, however, that patients who underwent segmentectomy had more locoregional recurrences, 11% versus 5%. The authors recommend segmentectomy as a standard surgical procedure for small peripheral NSCLC (early clinical stage IA).

Another large randomized multicenter trial, CALGB/ALLIANCE 140503, recently published the results of the comparison between lobectomy and sublobar resection. In this study, 687 patients with T1aN0 NSCLC were randomized to lobectomy (*n* = 357) and sublobar resection (*n* = 340), including wedge resection (*n* = 201). The authors reported that sublobar resection was noninferior to lobectomy; 5-year disease-free survival was 64.1% for lobectomy and 63.6% for sublobar resection, and recurrence-free survival was comparable (HR 1.05; 95% CI, 0.80 to 1.39). The 5-year disease-free survival was 71.2% with lobectomy versus 70.2% after sublobar resection.

Although anatomic segmentectomy has long been considered a compromising procedure performed for inadequate cardio-respiratory reserve, poor performance status, or in elderly patients, these two randomized controlled trials (JCOG and CALGB) demonstrated that overall and disease-free survival are comparable to those of lobectomy, and, for all these reasons, it is possible to state that, in the near future, anatomical segmentectomies (and, in selected cases, also wedge resections) will become the gold standard resection for patients with an early-stage NSCLC.

Furthermore, in recent decades, lung resection in non-intubated patients, the so-called “awake lung resection”, extended the possibility to also perform lung surgery in patients with a high number of comorbidities [31]. At the beginning, this type of surgery was used especially for surgical lung biopsies in patients with a high grade of suspicion of an interstitial lung disease. Then this technique was also used in secondary and primary lung tumors. At first, the surgeons performed wedge resections; then the awake resection was improved and segmentectomies and lobar resections were performed. The use of this technique is growing all around the world, although it is more technically demanding for the surgeon and for the anesthesiologist: the first has to make the lung resection in a ventilated lung, the second has to maintain a correct grade of sedation, to control the pain, the cough reflex (due to parenchymal manipulation), and the oxygen saturation in a non-intubated patient. Furthermore, in recent years these lung resections in “awake” patients are performed with the uniportal technique. Actually, a lung resection in a non-intubated patient performed in an uniportal way represents the ultimate minimally invasive thoracic surgery technique and, although it has a slower learning curve compared to a lung resection in an intubated patient, it is designed to grow more and more, and could also be a feasible option for “fit-for-surgery” patients. Unfortunately, randomized controlled trials are still lacking. 

In addition, the peri-operative outcomes of segmentectomies have been evaluated. Stamatis et al. [32] conducted a prospective, randomized, multicenter phase III study and found that segmentectomy was associated with lower peri-operative morbidity and appeared to offer a better quality of life compared with patients who underwent lobectomy. They enrolled a total of 108 patients with NSCLC < 2 cm, 54 patients undergoing lobectomy and 54 undergoing segmentectomy. Patients who underwent lobectomy 12 months after surgery showed deterioration in both physical (*p* < 0.001) and cognitive functions (*p* = 0.025) and pulmonary function such as dyspnea (*p* < 0.001) and fatigue (*p* = 0.003).

According to Harada and colleagues [33] less-extensive lung resections such as segmentectomies offer significantly better functional preservation (forced vital capacity (FVC) and forced expiratory volume in 1 s (FEV1)) than lobectomy at 2 and 6 months after surgery. The two randomized trials (JCOG and CALGB) also reported that the reduction in FEV1 at 6 and 12 months was greater in patients who underwent lobectomy.

Some authors reported a prolonged air leak with segmentectomy compared to lobectomy. The JCOG 0802 trial revealed an increased rate of air leak in the segmentectomy arm versus lobectomy, 6.5% and 3.4%, respectively (*p* = 0.04). Suzuki and associates [34] performed a randomized trial to evaluate the role of segmentectomy in comparison to lobectomy in patients with invasive peripheral NSCLC (T < 2 cm). Their work confirmed that sublobar resections are noninferior, with post-operative results comparable to those of lobectomy. However, in the group of patients who underwent segmentectomy, the authors noted an increased air leakage. Persistent air leakage during segmentectomy seems to be related to the use of electrocautery to divide the lung parenchyma during fissure completion [3,35], and the use of staplers to complete the intersegmental plane of the lung might reduce this complication.

## 5. Conclusions

VATS sublobar resections are increasingly becoming the standard indication for early-stage NSCLC. Nowadays, surgical techniques for segmentectomies (both simple and complex segmentectomies) are widely used worldwide. Before performing this kind of resection, it is mandatory to perform a careful pre-operative evaluation, including CT scan images, PET scanning, full pulmonary function tests, and a cardiac evaluation (according to the latest guidelines [7]). Moreover, both in the pre-operative evaluation period and in the intra-operative period, it is crucial to perform an assessment to establish oncologic margins and lymph node involvement (e.g., intra-operative frozen section). Minimally invasive surgical techniques such as video-assisted thoracic surgery (VATS) and robotic thoracic surgery (RATS) have also been shown to be safe procedures that guarantee oncologic outcomes comparable to those of “open” surgery and are also associated with less post-operative pain, shorter hospital stay, and faster recovery. In addition, robotic surgery with multiple degrees of freedom, 3D visualization, and reduced tremor can enable precise sublobar resection and precise lymphadenectomy.

In these patients, not only is the overall survival in early-stage NSCLC increased, but also the incidence of metachronous and contralateral lung cancer. Even if long-term survival still needs to be assessed, these parenchymal-sparing techniques have noninferior post-operative oncologic and pulmonary functional outcomes compared with lobectomy, but allow for more rapid recovery after surgery and do not exclude patients from other possible lung resections. Other advanced techniques, such as awake sublobar lung resections, allow surgical resection even in patients with high comorbidities. This recent surgical technique avoids the need for one-lung ventilation and general anesthesia, and allows operating on patients who, previously, would have been considered borderline for surgery.

Another important topic that several authors have addressed is spread through airspaces (STAS), which is considered a mode of lung cancer invasion. STAS is considered an independent risk factor for disease recurrence and poor overall survival with poor prognosis, and is found in 15–60% of patients with NSCLC [36]. Many publications on this topic claim that the surgical procedure affects the prognosis of NSCLC with STAS. Sublobar resection was associated with worse outcomes than lobectomy in patients with T1 NSCLC with STAS [37,38]. Ikeda et al. [39] recently published the results of a retrospective cohort study involving 555 patients (*n* = 148 with STAS and *n* = 407 without STAS) with stage IA NSCLC. This cohort study evaluated recurrence-free survival (RFS) and overall survival (OS) with wedge resection, segmentectomy, and lobectomy for pathologic stage non-small cell lung cancer IA with spread through air spaces. The authors found that, in patients with STAS, segmentectomy had comparable RFS and OS to those of the lobectomy group (5-year OS: segmentectomy 70.6% vs. lobectomy 72.6% *p* = 0.550; 5-year DFS: segmentectomy 92.9% vs. lobectomy 83.6% *p* = 0.213). Therefore, segmentectomy is a valid and appropriate surgical alternative to lobectomy in patients with stage IA NSCLC, with or without STAS.

Data from the two recent randomized trials suggest that segmentectomy may be a useful alternative to lobectomy for stage I NSCLC up to 2 cm. With increasing knowledge of the biology of non-small cell lung cancer, it may be useful to develop a clinicopathologic algorithm that incorporates patient pre-operative and intra-operative factors, tumor size, tumor histology, and segmental anatomy to better guide thoracic surgeons in a specific lung resection. Further, the advancement of radiologic software that provides unambiguous segmental imaging, in combination with the use of minimally invasive surgery, can help surgeons perform segmentectomies to spare lung parenchyma.

Finally, as mentioned earlier, lung cancer screening programs will increase the number of early-stage NSCLC diagnoses and thus increase the opportunities for sublobar lung resections.

## Figures and Tables

**Table 1:** Summary of randomized controlled trials (RCTs) comparing sublobar resection vs. lobectomy in non-small cell lung cancer.

Study	Year	Country	No Patients	NSCLC Stage	Inclusion of the Wedge Resection	Overall Survivall Sublobar Resection vs. Lobectomy	Recurrence Rate Sublobar Resection vs. Lobectomy
Lung Cancer Study Group	1995	USA	247	T1 N0 (peripheral tumor < 3 cm)	YES	Increased with lobectomy	Increased with sublobar resection
JCOG0802/WJOG4607L	2022	JAPAN	1106	IA (peripheral tumor ≤ 2 cm; consolidation-to-tumor ratio > 0.5 cm)	NO	Increased with segmentectomy	No difference in total relapse pattern
CALGB/Alliance 140503	2023	USA	697	T1aN0 (peripheral solid tumor ≤ 2 cm)	YES	Sublobar resection noninferior to lobectomy	No difference

**Table 2:** Simple and complex segmentectomy.

	Simple Segment	Complex Segment
Left Upper Lobe	Lingulectomy	
	Upper Division	
		1 + 2
		2
		3
Left Lower Lobe	6	
	Basal Pyramid	
		8
		9
		8 + 9
		9 + 10
		6 + 10
Right Upper Lobe		1 + 3
		1
		2
		3
Right Lower Lobe	6	
	Basal Pyramid	
		7
		8
		10
		8 + 9
		6 + 10
		9 + 10
